# Cutaneous Cryptococcosis Mimicking Leishmaniasis

**DOI:** 10.4269/ajtmh.17-0170

**Published:** 2018-01

**Authors:** Alexandre Moretti de Lima, Milena Marchini Rodrigues, Carmelia Matos Santiago Reis

**Affiliations:** 1 Department of Dermatology, Federal University of Mato Grosso do Sul (Universidade Federal do Mato Grosso do Sul - UFMS), Campo Grande, Brazil;; 2 Resident of the Dermatology Service, Hospital de Base, São José do Rio Preto State School of Medicine (FAMERP), São José do Rio Preto, Brazil;; 3 Dermatologist, Hospital Regional da Asa Norte (HRAN/SES), Brasília, Brazil

**Figure 1.**
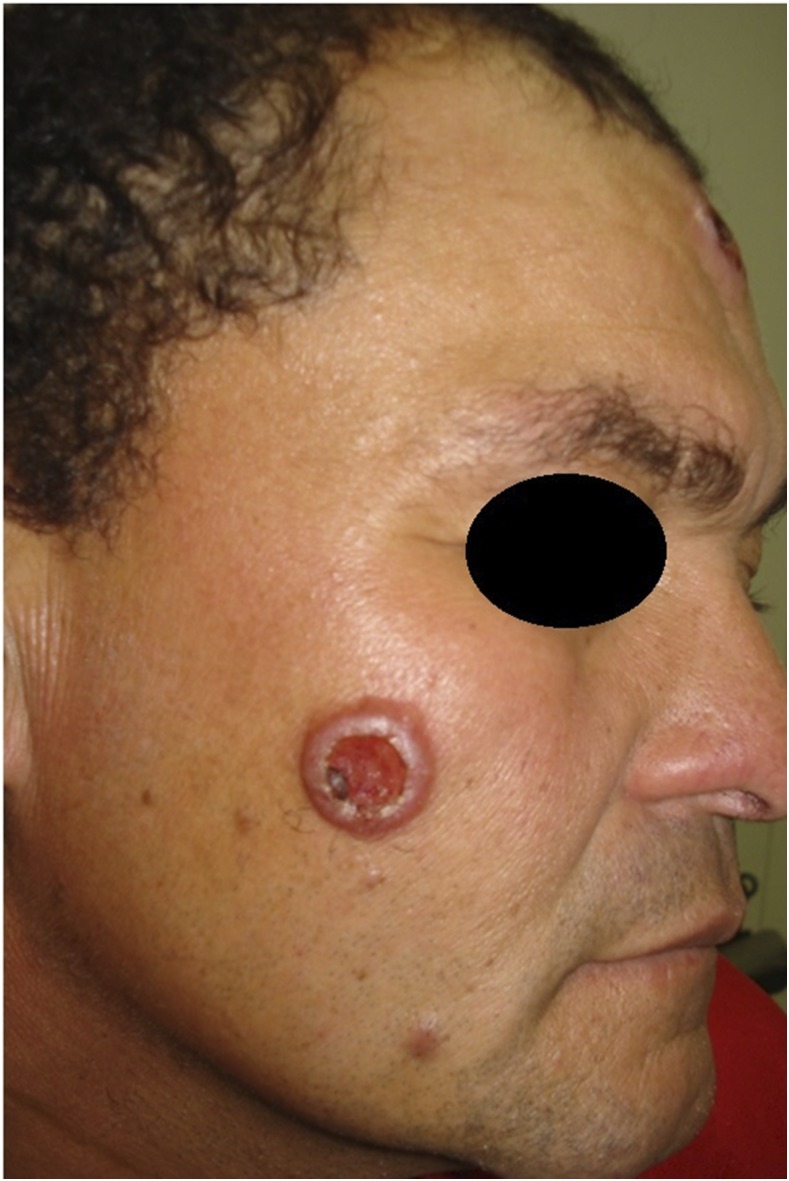
Ulcerated nodule with overlying crust on right malar region, 6 cm in size. This figure appears in color at www.ajtmh.org.

**Figure 2.**
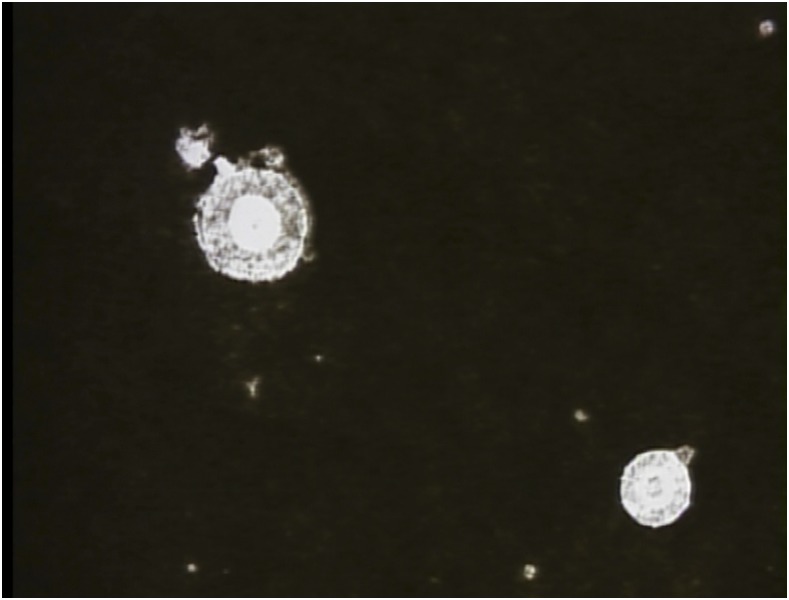
Sputum–direct examination for round structures compatible with *Cryptococcus* spp. (India ink, ×100). This figure appears in color at www.ajtmh.org.

**Figure 3.**
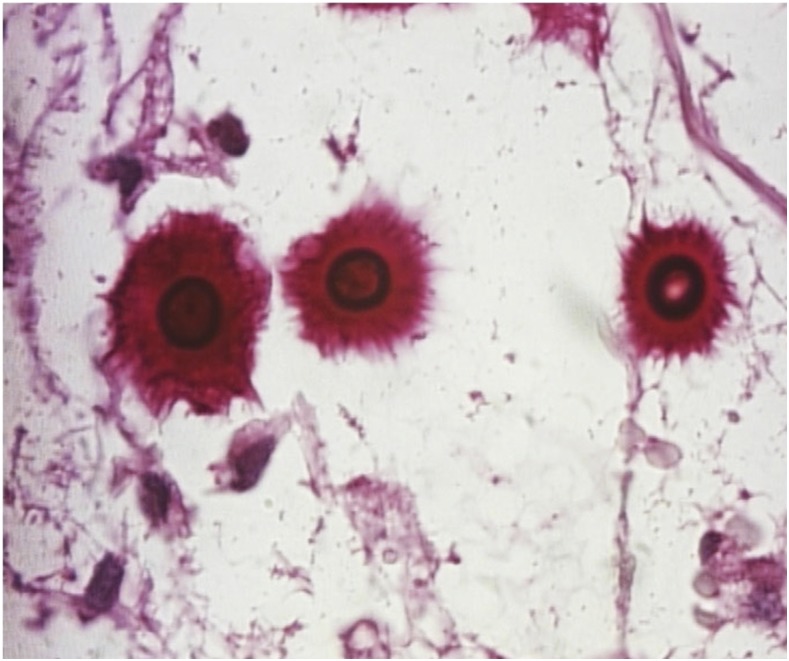
Rounded structures with thick capsules in the dermis, compatible with *Cryptococcus* spp. (Periodic acid-Schiff, ×400). This figure appears in color at www.ajtmh.org.

A 51-old-year male carpenter, who was a native of rural midwestern Brazil, presented with a 2-month history of a papule on the right malar region that had progressed to an ulcerated nodule. He had been diagnosed with Hansen’s disease 15 years prior and was under immunosuppressive therapy with prolonged courses of oral prednisone for treatment of his type 2 leprosy reaction. He had a history of environmental exposure to dense forests and areas of deforestation. Physical examination showed a 6-cm ulcerated nodule with an overlying crust on the right malar region, resembling leishmaniasis (Figure 1). His blood cultures and biochemical tests were normal, including serology for human immunodeficiency virus-1 and human immunodeficiency virus-2. Chest radiography revealed a nodule in the left lower lobe of the lung. Lumbar puncture was performed and routine parameters (cell counts, glucose, and protein) were within normal limits. Fungal culture and cerebrospinal fluid cryptococcal antigen were not available at our hospital. Direct microscopy with India ink revealed encapsulated forms typical of *Cryptococcus neoformans* (Figure 2). Histologic sample from the malar lesion showed rounded structures with thick capsules in the dermis, consistent with *Cryptococcus* species (Figure 3). Culture in sabouraud agar showed growth of *Cryptococcus* species, leading to a diagnosis of disseminated cryptococcosis involving lung and skin. The patient was treated with liposomal amphotericin B at a total dose of 4.5 g and then maintained on fluconazole 400 mg/week indefinitely, with total regression and healing of lesions.

Cryptococcosis occurs more commonly in immunocompromised hosts, including those with acquired immunodeficiency syndrome, Hodgkin’s lymphoma, or those who use immunosuppressive therapies including corticosteroids.^1–4^

*Cryptococcus neoformans* is a yeast-like encapsulated fungus, which is typically found in pigeon droppings.^1,2^ The main portal of entry is the respiratory tract, and lungs are the primary site of infection. In most instances, the infection is subclinical and self-limiting.^1,2^ However, the infection may progress by hematogenous dissemination to any part of the body.^1,2^ The skin is the most common site of dissemination, and cutaneous lesions occur in 5% to 15% of patients.^1–3^ The lesions may occur as ulcers, pustules, granulomata, abscesses, or herpetiform or molluscum contagiosum-like lesions.^3–5^ These skin lesions may simulate a variety of diseases, such as cellulitis, histoplasmosis, or basal cell carcinoma.^3–5^ In the case reported here, the lesion was an ulcerated nodule, mimicking cutaneous leishmaniasis. In addition to biopsies, the diagnosis may be made by other direct means such as with Tzanck smears, India ink, or potassium hydroxide examinations.^5^ Cryptococcosis can clinically mimic leishmaniasis, and the possibility that an ulcerated nodule in immunocompromised hosts may be caused by *C. neoformans* should be considered.
